# Hyperkalemia in patients treated with endoradiotherapy combined with amino acid infusion is associated with severe metabolic acidosis

**DOI:** 10.1186/s13550-018-0370-z

**Published:** 2018-02-27

**Authors:** Christian H. Pfob, Matthias Eiber, Peter Luppa, Florian Maurer, Tobias Maurer, Robert Tauber, Calogero D’Alessandria, Benedikt Feuerecker, Klemens Scheidhauer, Armin Ott, Uwe Heemann, Markus Schwaiger, Christoph Schmaderer

**Affiliations:** 1 Department of Nuclear Medicine, Technische Universität München, Klinikum rechts der Isar, Ismaningerstrasse 22, 81675 Munich, Germany; 2 Department of Nephrology, Technische Universität München, Klinikum rechts der Isar, Ismaninger Str. 22, 81675 Munich, Germany; 3 Hospital Pharmacy Department, Technische Universität München, Klinikum rechts der Isar, Ismaningerstrasse 22, 81675 Munich, Germany; 4 Department of Pathobiochemistry, Technische Universität München, Klinikum rechts der Isar, Ismaninger Str. 22, 81675 Munich, Germany; 5 Department of Urology, Technische Universität München, Klinikum rechts der Isar, Ismaningerstrasse 22, 81675 Munich, Germany; 6 Institute of Medical Statistics and Epidemiology, Technische Universität München, Klinikum rechts der Isar, Ismaningerstrasse 22, 81675 Munich, Germany

**Keywords:** Hyperkalemia, Radiotherapy, PRRT, RLT, Amino acid, Prostate cancer, Metabolic acidosis

## Abstract

**Background:**

Amino acid co-infusion for renal protection in endoradiotherapy (ERT) applied as prostate-specific membrane antigen (PSMA)-targeted radioligand therapy (RLT) or peptide receptor radionuclide therapy (PRRT) has been shown to cause severe hyperkalemia. The pathophysiology behind the rapid development of hyperkalemia is not well understood. We hypothesized that the hyperkalemia should be associated with metabolic acidosis.

**Results:**

Twenty-two patients underwent ERT. Prior to the first cycle, excretory kidney function was assessed by mercapto-acetyltriglycine (MAG-3) renal scintigraphy, serum biochemistry, and calculated glomerular filtration rate (eGFR). All patients received co-infusion of the cationic amino acids L-arginine and L-lysine for nephroprotection. Clinical symptoms, electrolytes, and acid-base status were evaluated at baseline and after 4 h.

No patient developed any clinically relevant side effects. At baseline, acid base status and electrolytes were normal in all patients. Excretory kidney function was normal or only mildly impaired in all except two patients with stage 3 renal insufficiency. All patients developed hyperkalemia. Base excess and HCO_3_^−^ were significantly lower after 4 h. In parallel, mean pH dropped from 7.36 to 7.29. There was a weak association between calculated (*r* = − 0.21) as well as MAG-3-derived GFR (*r* = − 0.32) and the rise in potassium after 4 h.

**Conclusion:**

Amino acid co-infusion during ERT leads to severe metabolic acidosis which induces hyperkalemia by potassium hydrogen exchange. This novel finding implies that commercially available bicarbonate solutions might be an easy therapeutic option to correct metabolic acidosis rapidly.

**Electronic supplementary material:**

The online version of this article (10.1186/s13550-018-0370-z) contains supplementary material, which is available to authorized users.

## Background

Endoradiotherapy (ERT) is an emerging therapeutic procedure in advanced solid tumors. In particular, prostate-specific membrane antigen (PSMA)-targeted radioligand therapy (RLT) and peptide receptor radionuclide therapy (PRRT) utilizing radiolabeled peptides are used to control advanced-stage prostate [1] and neuroendocrine cancer [2, 3]. PRRT is also used for glomus tumors and meningiomas that overexpress somatostatin receptor subtype II [4–7].

Therapeutic agents in current use for PSMA-targeted RLT and PPRT exhibit high renal excretion rate [8, 9]. Especially radiolabeled PRRT peptides are filtered by the glomerulus and effectively taken up by proximal tubule cells [10, 11] via megalin-receptor-mediated endocytosis [12, 13]. After uptake, the peptide is translocated to the lysosomal apparatus for further handling [14]. Retention at the proximal tubule causes a high effective dose of radiation to the kidneys; thus, there is a need for inhibiting renal uptake of labeled peptides to limit the amount of radioactivity to renal structures. Otherwise, the potential radiation-induced nephropathy could limit effective single and cumulative dose and the number of treatment cycles.

Previous publications have reported a reduced renal dose by co-infusing the positively charged amino acids (AAs) L-lysine and/or L-arginine for kidney protection during PRRT therapy [10], a practice that has entered current guidelines [7]. Recommendations of PRRT have been transferred to PSMA-targeted RLT due to the high uptake of the radiopeptide in the kidney. Mechanistically, AAs bind to the tubular megalin/cubulin system and compete with the radiopeptide for reabsorption [11]. For PRRT, a 15 to 60% reduction in renal uptake is reported [2, 10, 11, 15, 16].

However, co-infused AA can cause adverse effects such as vomiting, nausea, and hyperkalemia [10, 15, 17–19]. The levels of hyperkalemia that have been reported in studies are potentially life-threatening and ask for close monitoring and rapid correction. However, so far, the cause of the hyperkalemia remains unclear. It has been shown that there is a potassium shift from the intra- to the extracellular compartment caused by cationic AA [10, 15, 20, 21].

It is well known that there is a close relationship between potassium and the acid base status in the peripheral blood as acidosis causes decreased potassium secretion and increased reabsorption in the collecting ducts. Furthermore, an inverse correlation between the potassium concentration and the pH in plasma is well known. Therefore, we hypothesized that AA infusion for nephroprotection in ERT might lead not only to hyperkalemia but also to a substantial alteration of the acid base status namely metabolic acidosis. To the best of our knowledge, no data are published investigating the acid base status after AA co-infusion during ERT. A potential correlation could have direct clinical implications as acidosis can be easily corrected by commercially available, inexpensive bicarbonate infusions or oral bicarbonate therapy.

## Methods

### Patients

A total of 22 patients underwent 10 cycles of PRRT and 12 cycles of RLT, respectively. Twelve patients had histopathologically confirmed prostate cancer, one had thyroid cancer, and nine had neuroendocrine tumors. Patient characteristics are summarized in Table 1.

**Table 1:** Clinical characteristics for each patient before endoradiotherapy with amino acid co-administration

Patient	Primary tumor	ERT	Sex	Age (a)	Weight (kg)	Height (m)	GFR (CKD-EPI)	KDIGO Grade of CKD	MAG 3 clearance ml/min/1.73 qm BSA	Creatinine (mg/dl)	BUN (mg/dl)
1	NET	PRRT	F	45	50	1.62	105	G1	236	0.7	14
2	NET	PRRT	M	57	58	1.71	74	G2	195	1.0	21
3	Prostate cancer	RLT	M	66	74	1.76	93	G1	178	0.8	16
4	Prostate cancer	RLT	M	76	90	1.84	83	G2	178	0.9	14
5	Prostate cancer	RLT	M	64	71	1.74	90	G1	252	0.9	17
6	Prostate cancer	RLT	M	73	75	1.74	60	G2	157	1.2	13
7	Prostate cancer	RLT	M	62	85	1.78	91	G1	269	0.9	13
8	Prostate cancer	RLT	M	73	89	1.75	74	G2	175	1.0	13
9	NET	PRRT	M	63	130	1.72	91	G1	253	0.9	21
10	NET	PRRT	M	71	102	1.84	60	G2	187	1.2	12
11	Prostate cancer	RLT	M	72	67	1.74	67	G2	175	1.1	28
12	NET	PRRT	F	54	63	1.76	84	G2	257	0.8	14
13	Prostate cancer	RLT	M	77	64	1.71	72	G2	224	1.0	18
14	Prostate cancer	RLT	M	76	66	1.89	92	G1	143	0.7	14
15	Prostate cancer	RLT	M	73	84	1.84	74	G2	208	1.0	17
16	Prostate cancer	RLT	M	57	87	1.90	112	G1	251	0.6	12
17	NET	PRRT	M	69	95	1.86	76	G2	233	1.0	20
18	NET	PRRT	M	73	86	1.80	60	G2	132	1.2	21
19	NET	PRRT	M	53	80	1.76	86	G2	211	1.0	17
20	NET	PRRT	M	59	85	1.77	55	G3	122	1.4	15
21	Prostate cancer	RLT	M	73	78	1.80	49	G3	129	1.4	32
22	Thyroid	PRRT	F	62	52	1.57	93	G1	192	0.7	9
min.				45	50	1.57	49		122	0.6	9
max.				77	130	1.90	112		269	1.4	32
mean				66	79	1.77	79		201	1.0	17
median				68	79	1.76	80		195	1.0	16
SD				08	17	0.08	16		43	0.2	5.3

*NET* Neuroendocrine tumor, *GFR* glomerular filtration rate, *CKD-EPI* Chronic Kidney Disease Epidemiology Collaboration, *MAG3* mercapto-acetyltriglycine, *KDIGO* Kidney Disease—Improving Global Outcomes, *BUN* Blood urea nitrogen

All patients received a standard dose of 1000 ml 2.5% Arg/Lys solution (details for production and composition are included in Additional file 1) with a constant flow rate of 250 ml/h over 4 h starting 30 min prior to ERT. A 5-HT3 antagonist (3 mg granisetron) was co-injected to prevent nausea and vomiting in patients with PRRT therapy and 5-HT3 antagonist (1 mg granisetron) in combination with a corticosteroid (8 mg dexamethasone) in patients with PSMA-targeted RLT. The use of AA and radiopeptides complied with the German Medicinal Products Act [22], AMG §13 No. 2b and were reported to the regulatory office (Regierung von Oberbayern).

The retrospective analysis of patient data was approved by the Ethics Committee and Institutional Review Board of Klinikum rechts der Isar of the Technical University Munich (permit 157/17 s) and is in accordance with the 1964 Helsinki declaration and its later amendments or comparable ethical standards. All patients provided signed informed consent for treatment and publication of their data.

### Scintigraphic and laboratory evaluation

Twenty-one patients underwent a renal function scintigraphy using mercapto-acetyltriglycine (MAG3) prior to the first cycle. 99mTc-MAG-3 was injected as a slow intravenous bolus shortly before start of a multiphase dynamic acquisition (vascular phase, functional uptake, cortical transit, and excretion phase). Two plasma samples were taken for estimation of MAG-3-derived GFR.

Within 24 h before AA application, glomerular filtration rate (eGFR, according to the Chronic Kidney Disease Epidemiology Collaboration (CKD-EPI) [23]), albumin, creatinine, blood urea nitrogen (BUN), phosphate, potassium, sodium, base excess, anion gap, bicarbonate, and chloride were assessed by serum and blood gas analytics. Four hours after the start of the AA infusion, blood gas analysis samples were re-collected. Blood samples were collected into serum (Serum-Monovette) and lithium heparin gel tubes (S-Monovette). Application of the tourniquet was as brief as possible to minimize false-positive potassium values due to hemolysis. Within 30 min of the blood drawing, blood samples were centrifuged at 1830×*g* (Multicentrifuge 3SR+, Thermo Scientific) for 5 min. Serum potassium levels were measured with a Cobas 8000 indirect ion-sensitive electrode system (Roche Diagnostics, Mannheim, Germany). All samples were screened for hemolysis as follows. Absorbance of the diluted (1:26) serum samples was measured at 570 nm (primary wavelength) and 600 nm (secondary wavelength), and hemolysis indices were calculated according to the manufacturer’s instructions. The indices ranged from 3.7 to 4.7 mmol/l. Whereas hyperkalemia is routinely defined by our laboratories as serum potassium levels of > 5.0 mmol/l, severe hyperkalemia was arbitrarily defined as serum potassium values of ≥ 6.0 mmol/l.

The analysis of standard HCO_3_^−^ and base excess (BE) and measurement of serum potassium were performed on a Rapid-Lab 1265 blood gas analyzer (Siemens Medical Solutions, Eschborn, Germany) with automated sample delivery, parameter calibration, and daily quality control checks. The analyzer included electrochemical sensors for measuring pH, pO_2_, pCO_2_, sodium, potassium (mmol/l), chloride, ionized calcium, glucose, and lactate. HCO_3_^−^ was calculated using the Henderson-Hasselbalch equation: HCO_3_^−^ (mmol/l) = 0.03037 × pCO_2_ × 10^pH−6.105^. The BE was calculated as BE (mmol/l) = (1 − 0.014 × ctHb) × [(HCO_3_^−^ − 24.8) + (1.43 × ctHb + 7.7) × (pH − 7.40)].

### Statistical analysis

All continuous data reported are expressed as mean, median, standard deviation, and range. Two-sample *t* tests were used to evaluate differences between individual groups. Changes in the hyperkalemia-associated parameters were evaluated using paired *t* tests. Because of their symmetric distributions, normality of the differences was assumed. To calculate correlations between single factors, Pearson’s correlation coefficient was performed. A significance level of *α* = 5% was used. Statistical analyses were conducted using GraphPad Prism (version 7.00, 2014; GraphPad Software, La Jolla California USA) and R (version 3.3.2; R Foundation, Vienna, Austria; https://www.r-project.org).

## Results

### Side effects of the AA infusion

No clinically relevant side effects as nausea and vomiting, palpitations, or general malaise have been reported by the patients during and after 2.5% Arg/Lys amino acid infusion, and we observed no adverse events (CTCAE grade 3, 4, or 5).

### Renal function

As reduced kidney function is a significant risk factor for the development of hyperkalemia, we first grouped patients according to the Kidney Disease—Improving Global Outcomes (KDIGO) CKD stages (eGFR calculated according to the CKD-EPI formula and graded after KDIGO CKD stage grading 2012) [23]. In only two patients, we found a significantly reduced GFR according to KDIGO Grade G3. The remaining patients had normal or only mildly impaired renal function (Table 1). A complete overview of renal function parameters before therapy is given in Table 2.

**Table 2:** Changes in potassium and acid base parameters before and after endoradiotherapy with amino acid co-infusion

	Unit	Normal range	Baseline			4 h			*P* value (paired *t* test)
			Mean	SD	Absolute number and % out of the normal range	Mean	SD	Absolute number and % out of the normal range	
Potassium (serum)	mmol/l	3.5–5.0	4.52	0.33	1 (4.17%)	6.14	0.52	24 (100%)	< 0.0001
Potassium (blood gas analysis)	mmol/l	3.5–5.3	4.25	0.32	0 (0.00%)	5.80	0.43	20 (83.33%)	< 0.0001
Base excess	mmol/l	-2 - 3	− 0.55	2.23	9 (37.50%)	− 5.80	1.96	23 (95.83%)	< 0.0001
pH		7.350–7.450	7.36	0.05	9 (37.50%)	7.29	0.04	24 (100%)	< 0.0001
HCO3^−^	mmol/l	21–26	23.42	1.76	2 (8.33%)	19.07	1.76	22 (91.67%)	< 0.0001
Chloride	mmol/l	98–106	103.8	3.5	6 (25.00%)	109.9	3.3	20 (83.33%)	< 0.0001
Anion gap	mmol/l	10–20	14.6	2.6	3 (12.50%)	10.9	2.7	9 (37.50%)	< 0.0001

### Comparison of lab tests before and 4 h after the start of ERT with AA co-infusion

#### Potassium

##### Normal potassium levels before amino acid infusion

All patients showed normal serum potassium levels with a mean of 4.47 mmol/l (median 4.45 mmol/l, SD 0.30 mmol/l, and range 4.0–5.0 mmol/l) in the routine biochemistry measurement and with a mean of 4.21 mmol/l (median 4.20 mmol/l, SD 0.29 mmol/l, and range 3.8–4.9 mmol/l) in the blood gas analysis at baseline (Table 2).

##### All patients developed hyperkalemia after AA infusion

**Fig. 1:**
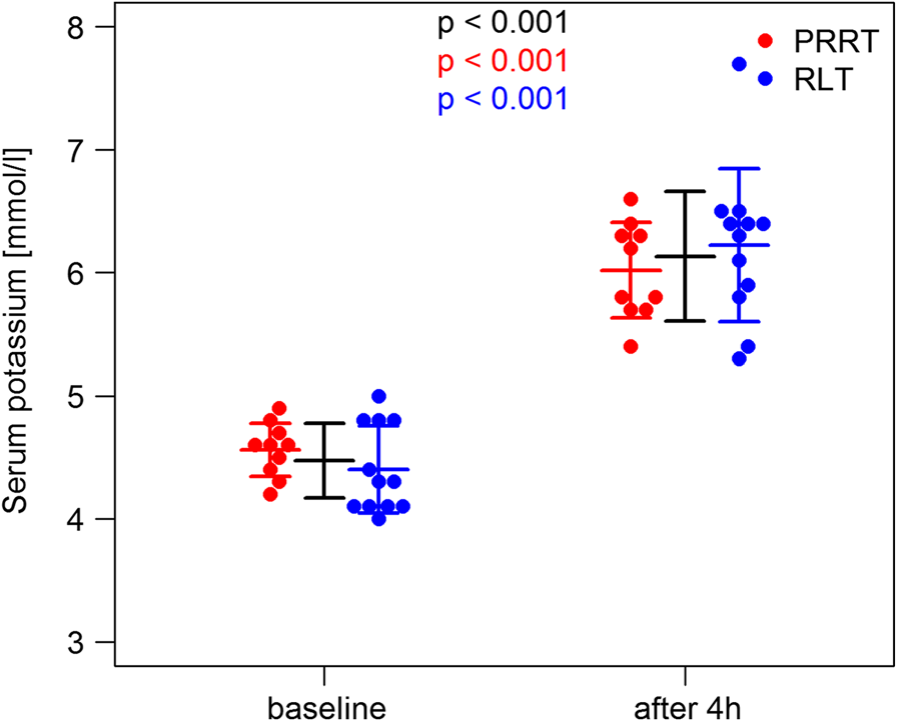
Significant increase in potassium after ERT and 2.5% Arg/Lys amino acid co-infusion (Cobas Analyzer)

**Fig. 2:**
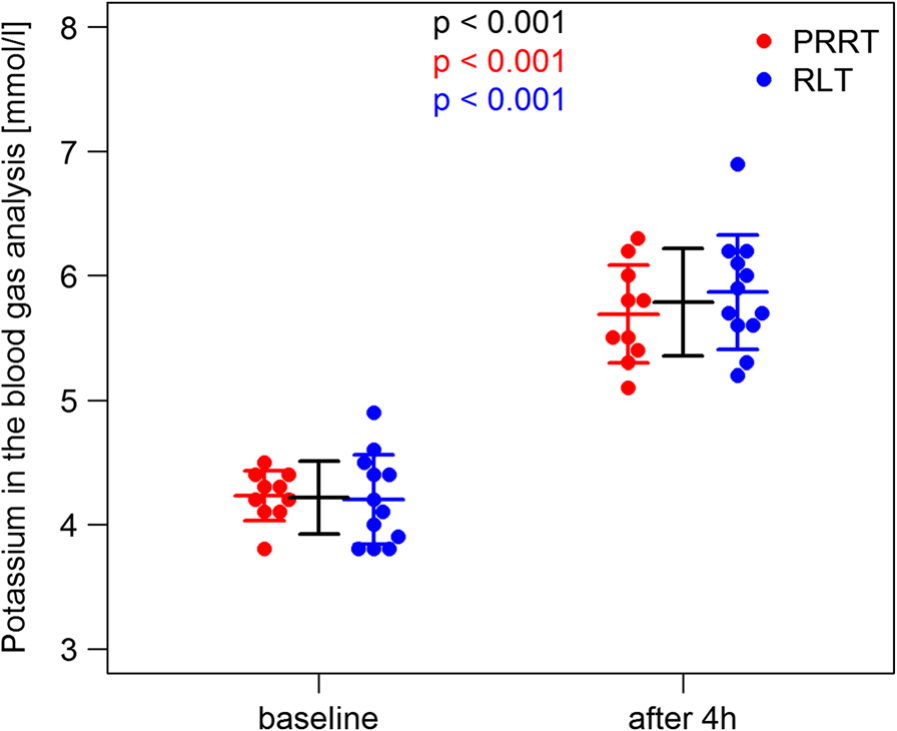
Significant rise in potassium after ERT and 2.5% Arg/Lys amino acid co-infusion (blood gas analysis)

The mean serum potassium level increased in all patients to a mean of 6.13 mmol/l (6.25 mmol/l, SD 0.52 mmol/l, and range 5.3–7.7 mmol/l) resulting in an average absolute increase of 1.66 mmol/l. The mean potassium measured on the blood gas analyzer was 5.79 mmol/l (median 5.75 mmol/l, SD 0.42 mmol/l, and range 5.1–6.9 mmol/l) with serum values ≥ 6.0 mmol/l in 13/22 cycles (59.09%) and ≥ 7.0 mmol/l in 1/22 cycles (4.55%) with 7.7 mmol/l (Figs. 1 and 2). The changes in biochemical parameters relevant for acid base and electrolyte disorders (base excess, pH, HCO3^−^, chloride, anion gap) are presented in Table 2.

##### Weak correlation between baseline kidney function and increase in potassium

**Fig. 3:**
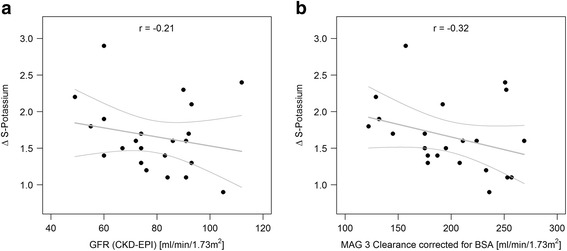
Weak correlation of calculated GFR (**a**) and MAG3-derived GFR (**b**) with Δ serum potassium

We observed a weak relationship between the rise in potassium after AA infusion and the calculated (Fig. 3a; *r* = − 0.21) and the MAG3-derived GFR (Fig. 3b; *r* = − 0.32).

#### pH level and anion gap decrease parallel development of hyperkalemia

In 21/22 therapeutic cycles, the patients’ blood pH levels decreased; in 1/22 cycle, the patients’ blood pH increased by 0.008. In all cycles, patients’ blood pH levels changed from a mean of 7.36 (median 7.38, SD 0.05, and range 7.26–7.45) at baseline to a mean of 7.29 (median 7.29, SD 0.03, and range 7.22–7.35) at 4 h after beginning AA administration.

The anion gap decreased from 14.7 (median 14.8, SD 2.6, and range 9.2–20.2) to 11.1 (median 11.6, SD 2.6, and range 7.2–17.8) 4 h after therapy which was still in the normal range.

#### Base excess and HCO_3_^−^

**Fig. 4:**
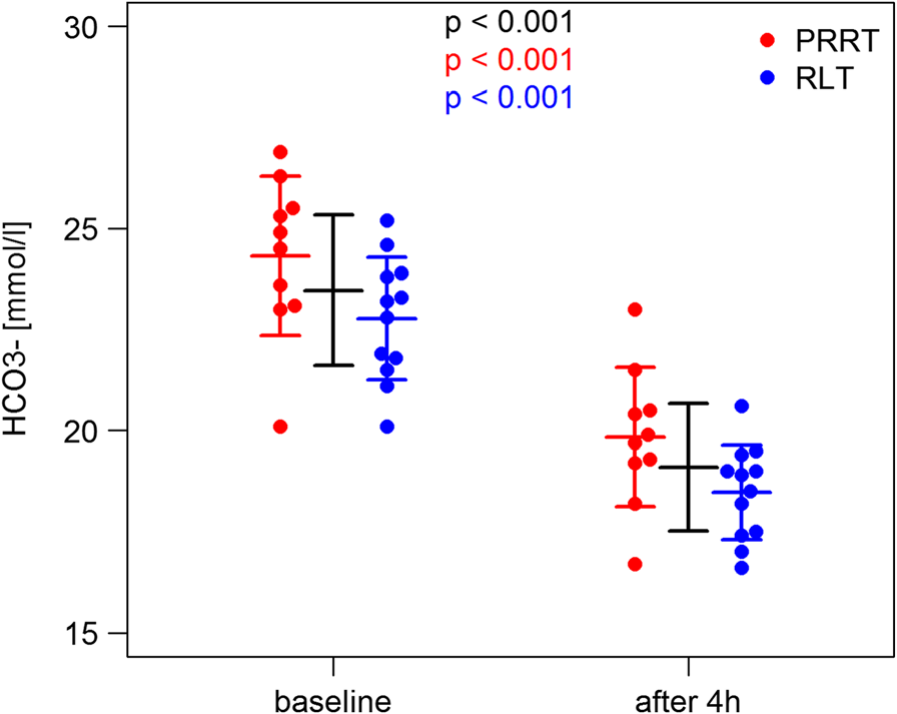
Significant decrease in bicarbonate (HCO3^−^) after ERT and 2.5% Arg/Lys amino acid co-infusion

**Fig. 5:**
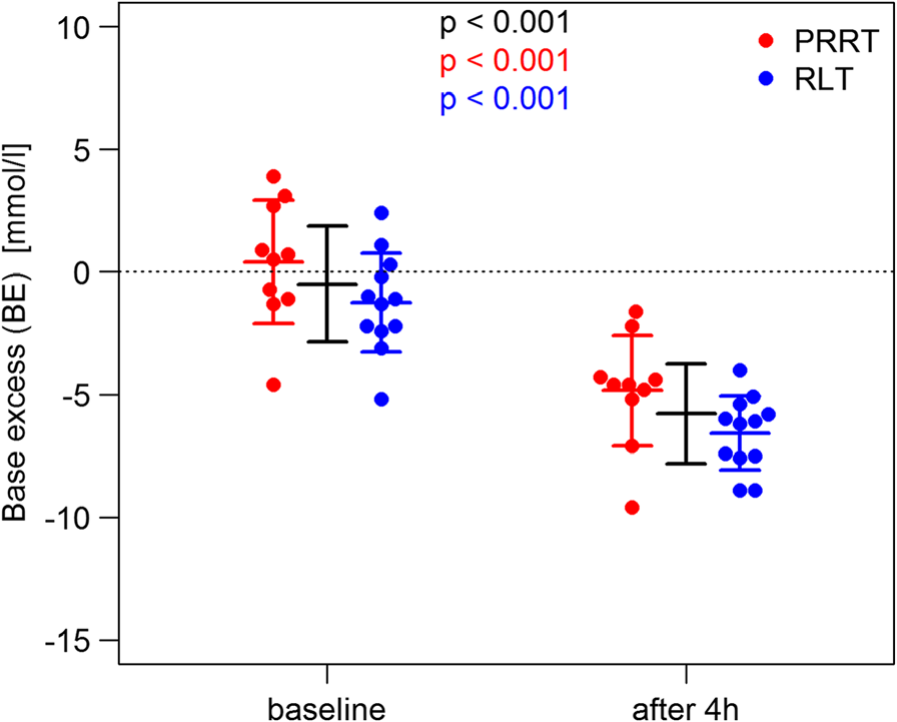
Significant drop in base excess after ERT and 2.5% Arg/Lys amino acid co-infusion

We found also severe changes in bicarbonate and base excess serum levels (Figs. 4 and 5). At baseline, mean BE was − 0.49 (median − 0.85, SD 2.30, and range − 5.2–3.9). Four hours after the start of the amino acid application, patients had a decreased BE with a mean of − 5.79 (median − 5.6, SD 1.99, and range − 9.6 to − 1.6). The baseline HCO_3_^−^ mean was 23.47 (median 23.45, SD 1.83, and range 20.1–26.9), which decreased 4 h after the start of the amino acid administration to a mean of 19.07 (median 19.09, SD 1.54, and range 16.6–23) in all patient cycles. The mean change of HCO_3_^−^ was − 4.38. Table 2 summarizes these findings.

## Discussion

To the best of our knowledge, this is the first study that measured acid-base status together with potassium levels, clinical relevant side effects, and renal function in patients treated with RLT or PRRT and simultaneous AA infusion for renal protection. The main findings are simultaneous AA infusion for renal protection in ERT (1) causes severe hyperkalemia, (2) does not lead to clinically apparent side effects, and (3)—as the most relevant finding—causes severe metabolic acidosis.

In line with recent reports, we observed hyperkalemia as a major pharmacological side effect of the arginine-lysine solution [10, 15, 17–19]. The use of an AA solution for renal protection is based on its competition with the radiopeptide for receptor-mediated uptake in tubular kidney cells [10, 16, 20]. The megalin receptor is a multi-ligand receptor and plays an important role for renal proximal tubule reabsorption of somatostatin analogues and different other proteins and trace elements [12]. Megalin-deficient mice showed less uptake in the renal cortex in autoradiography [24]. Melis et al. could demonstrate renal uptake of many radiopeptides (e.g., lutetium-labeled octreotate) in rats and reduced uptake after co-administration of amifostine. The latter is used as an effective cytoprotective agent in cancer therapy to prevent side effects [13, 21]. Despite the lack of preclinical data on inhibition of renal uptake for peptides used for PSMA-RLT, AA co-administration was used in all patients included in this analysis. This clinical decision was based on the low toxicity and theoretical protective effects as PSMA is well known to undergo clathrin-dependent internalization—an effect also known for the megalin receptor in kidney tubular cells [25, 26].

First reports on efflux of potassium from cells to the extracellular compartment in association with the administration of cationic AA like L-arginine and L-lysine date back as early as 1964 [25–29]. In commercially available AA solutions, acidosis is listed as a common side effect. In one study, a drop in bicarbonate after 4 h and more significantly after 10 h has been reported after amino acid infusion as co-medication in ERT [15]. However, this report lacks complete blood gas analysis, so it can only be assumed that the potassium shift and loss of buffer bases went along with metabolic acidosis in these patients.

In principle, metabolic acidosis can develop in three different ways: (1) addition of exogenous acids (in this case either infusion of cationic AA or the acidity of the AA infusion itself), (2) accumulation of hydrogen ions as result of inadequate renal excretion due to chronic renal insufficiency, and (3) increased production of endogenous acids (e.g., lactic acidosis or ketoacidosis). Notably, measured, calculated kidney function and acid base status has been normal or only mildly impaired in the majority of our patients before AA infusion. Therefore, it is unlikely that a significant reduction in excretory kidney function was the main reason for the accumulation of H^+^ ions. The mild renal insufficiency we found in more than 60% of patients should not lead to the development of metabolic acidosis in these early stages [30, 31]. In addition, there is no indication that production of endogenous acids is increased as the patients did not develop any clinical symptoms of hypoperfusion. This can be, e.g., be found in septic patients leading to the production of lactic acid, nor diabetic ketoacidosis or any other clinical state and consequently to the production of endogenous acids. The most plausible reason for the development of acidosis is the addition of exogenous acids. It is well known that total parenteral nutrition containing cationic acids can cause metabolic complications, one of the most common metabolic acidosis [27, 32]. This is in part caused by cationic AA (e.g., L-arginine and L-lysine) which can release H^+^ ions into the extracellular space. A clear hint in our study towards this mechanism is the finding of a decrease in serum pH. The H^+^ ions are buffered by the bicarbonate buffer leading to a significant reduction in bicarbonate which is also in line with the data reported here. Extracellular hydrogen ions are buffered intracellularly in exchange for potassium which is driven to the extracellular space. We detected metabolic acidosis and severe hyperkalemia shortly after amino acid infusion of cationic ions. It is known that ketogenic AAs as lysine are metabolized to ketones [33]. Accumulation of ketones leads to ketoacidosis. But the finding of a normal anion gap does not support the development of ketoacidosis [29]. It is more probable that H^+^ ions are generated directly by cationic amino acids [27].

The pH of the AA solution was adjusted to the range between 6.3 and 6.5 to reduce pharmacological side effects. Nevertheless, it is still an acidic solution and could reduce the pH of the peripheral blood. This is regulated in healthy patients with elimination of hydrogen ions by secretion in the tubular lumen of the kidney [28]. This process takes hours to be effective so a fast mechanism is needed if the body is faced with large acid loads. Therefore, cells (especially muscle cells) take up hydrogen ions and keep electroneutrality shift potassium in the serum [34]. This is the same mechanism as described above for infusion of AA, but this shift is caused not by cationic AA (which are consecutively also metabolized to H^+^ ions), but free hydrogen ions. Furthermore, renal excretion of hydrogen ions is impaired if metabolic acidosis is present [35]. Due to the partly severe hyperkalemia 4 h after the beginning of AA administration, we were not able to observe the potassium levels without treatment for more than 4 h. We have started individual treatments to normalize potassium level, e.g., suing sodium bicarbonate solution immediately after the results of the 4-h control blood samples were available.

As acid-base disorders and potassium handling are highly related, it is unclear what is the initial event: hyperkalemia causing H^+^ outflux or metabolic acidosis causing hyperkalemia. As our patients have been normokalemic at the start of the therapy and have developed hyperkalemia after 4 h, the most common reasons for the development of hyperkalemia can be ruled out: chronic kidney disease (which has been either absent or mild in our patients), pharmacological therapy like potassium substitution (not present, AA used during therapy does not contain potassium), or Renin-Angiotensin-System Blockade (not present). In addition, the elevation of serum potassium is highly unlikely to be related to death of tumor cells as the effect of ERT can be measured only after days and weeks, not hours.

Considering clinical side effects, we found that the arginine-lysine solution was very well tolerated. No patient complained of any known side effects of AA administration, while in studies by Lapa et al. and Rollemann et al., vomiting, palpitations, chest pain, and general malaise [10, 18, 19] were reported. Vomiting can be caused as a direct effect of the AA solution which has been described to cause a delay in gastric emptying and lowers the esophageal spincter tone [36]. The exact method of manufacturing of the AA mixture was only described by Rolleman et al.in detail, and the protocol differs from the one used in our in-house pharmacy. Thus, the difference in side effects could be related to differences between the mixtures.

Concerning hyperkalemia, vomiting itself should be considered as a possible influencing factor, as vomiting causes loss of acid and can cause metabolic alkalosis. Therefore, theoretically severe vomiting could protect from hyperkalemia during amino acid infusion. In practice, the reported numbers of patients having the side effect of vomiting in other studies is very low. In general, this is unlikely to be the sole explanation and specially in our patient cohort lacking clinically significant vomiting this relation can be excluded. It must be noted that—even if to date no serious cardiovascular events have been reported—the level of potassium reached with the amino acid infusion is potentially harmful or even lethal and cannot be ignored, even if the elevation is only transient [15]. Finally, tumor lysis caused by ERT itself cannot be ruled out as the cause of hyperkalemia (and in consequence metabolic acidosis by potassium shift into the cells in exchange with H^+^ ions) completely but seems very unlikely. As we lack a control group which did not get an AA co-infusion, this question remains open.

## Conclusions

The co-infusion of arginine and lysine solution during ERT is clinically well tolerated but leads to severe hyperkaliemia in a majority of patients. We have shown here that hyperkalemia is paralleled by severe metabolic acidosis most likely caused by increased hydrogen production through the infusion of cationic amino acids and potassium shift to the extracellular space. However, our data lack final proof on the sequence of these changes. Prospective clinical trials with serial measurements of electrolytes and acid-base status during the AA or placebo infusion in patients undergoing ERT are warranted to address this issue. However, we are convinced that our data back the approach to treat patients with oral or i.v. bicarbonate to correct acidosis and subsequently hyperkalemia very early during the AA infusion. Bicarbonate administration might even be considered prior to AA infusion to load bicarbonate buffer before hydrogen challenge.

## Additional file

Additional file 1: Composition and production of arginine-lysine solution. (DOCX 13 kb)
