# Supplementary material for: Hyperkalemia in patients treated with endoradiotherapy combined with amino acid infusion is associated with severe metabolic acidosis
Source: EJNMMI Res. 2018 Feb 27;8:17. doi: 10.1186/s13550-018-0370-z (PMC5829280; doi:10.1186/s13550-018-0370-z)
Supplement: Supplementary file 1 — Composition and production of arginine-lysine solution. (DOCX 13 kb) [file 13550_2018_370_MOESM1_ESM.docx]

**Additional file**

**Composition and production of arginine-lysine solution**

One liter of the solution was composed of arginine-HCl 30.24 g (equivalent to 25.0 g arginine, Fagron, Rotterdam, The Netherlands), lysine-HCl 31.25 g (equivalent to 25.0 g lysine, Fagron) and water for injection 953.51 g. The solution was adjusted to pH 6.3 to 6.5 with 15% NaOH (Merck, Darmstadt, Germany). All components conformed to the European Pharmacopoeia. The solution was produced under GMP conditions (cleanroom class A in C) in our hospital pharmacy. Arginine and lysine were dissolved in water for injection, filter-sterilized (PALL, Dreieich, Germany) and stored in prewashed sterile infusion bottles (Glass type 1, SGD, Mers-les-Bains, France). Bottles were sealed with bromobutyl caps (West Pharmaceuticals, Eschweiler, Germany) and capped. Filling and sealing was performed in a grade A environment with a grade C background. Sterile filtrated medical argon (Sauerstoffwerke Friedrichshafen, Friedrichshafen, Germany) was used to create a protective atmosphere during the filling process. Sealed bottles were autoclaved for 15 min at 121°C and tested for sterility, visible and sub-visible particles, endotoxins, pH, concentration, and tonicity.
